# Challenges in IgA Nephropathy Management: An Era of Complement Inhibition

**DOI:** 10.1016/j.ekir.2023.06.010

**Published:** 2023-06-21

**Authors:** Vladimir Tesař, Jai Radhakrishnan, Vivek Charu, Jonathan Barratt

**Affiliations:** 1 Department of Nephrology, Charles University, Prague, Czech Republic; 2 Columbia University Medical Center, New York, New York, USA; 3 Department of Pathology, Stanford University, Palo Alto, California, USA; 4 Department of Cardiovascular Sciences, University of Leicester, Leicester, UK

**Keywords:** alternative pathway, biomarkers, clinical trials, complement inhibition, IgA nephropathy, lectin pathway

## Abstract

IgA nephropathy (IgAN) is the most common glomerular disease worldwide, with an estimated annual incidence of 25 per million adults. Despite optimized supportive care, some patients fail to achieve disease control and suffer progressive deterioration of kidney function. In this subpopulation of patients, the Kidney Disease: Improving Global Outcomes 2021 guidelines recommend consideration of corticosteroids; however, their use is associated with significant side effects. Ongoing clinical trials are expected to identify corticosteroid-sparing therapies to help improve treatment and prognosis for patients with IgAN. It has been well-documented that the complement system plays a significant role in IgAN pathogenesis, and several complement inhibitors are now entering late-stage clinical development. This review evaluates what we know about the role of complement in the pathophysiology of IgAN and considers how the availability of targeted complement inhibitors may impact future clinical practice. Key knowledge gaps are evaluated, and research opportunities are recommended to help guide clinical decision-making and optimize patient outcomes. Such gaps include evaluating the relative contribution of the alternative and lectin pathways to disease pathogenesis, and the importance of determining the dominant pathway driving IgAN progression. Continued research into the staining of complement proteins in kidney biopsies and identifying targeted biomarkers to assess disease progression and treatment responses will also be needed to support the implementation of newer therapies in clinical practice. Considering the future horizons for enhancing the care of patients with IgAN, tackling the outstanding challenges now will help prepare for the best possible future outcomes.

IgAN is the most common primary glomerular disease worldwide.^1^^,^^2^ Of the estimated 25 cases per million people each year globally, incidences are higher in children and young adults (20–30 years of age) than in the elderly (>65 years of age).^1^^,^^3^ The epidemiology of IgAN varies by country or region, and the gender distribution also differs geographically, being more common among males than females in North America and Europe, with an even distribution observed in East Asia.^4^^,^^5^ IgAN is most prevalent in Asians, followed by Caucasians, and infrequently occurs in Africans.^4^ The clinical course of IgAN ranges from isolated asymptomatic microscopic hematuria to chronic proteinuria with a decline in kidney function and, rarely, rapidly progressive glomerulonephritis.^5^^,^^6^ Overall, IgAN is a leading cause of chronic kidney disease, with 10% to 40% of patients having kidney failure within 10 to 20 years of diagnosis.^7^^,^^8^ Initial management of patients with IgAN focuses on providing optimized supportive care, including lifestyle advice, blood pressure management using a maximally tolerated dose of renin-angiotensin-aldosterone system inhibitors, and addressing cardiovascular risk.^9^ However, some patients cannot achieve control of proteinuria and continue to be at high risk of kidney failure over time.^10^ Sodium-glucose cotransporter-2 inhibitors are emerging as a treatment option in IgAN. Recently, a prespecified analysis from the Dapagliflozin and Prevention of Adverse Outcomes in Chronic Kidney Disease trial demonstrated significantly reduced risk of chronic kidney disease progression in patients with IgAN treated with dapagliflozin added to renin-angiotensin-aldosterone system inhibitors.^11^ Beyond maximal supportive care, Kidney Disease: Improving Global Outcomes 2021 guidelines recommend that patients at high risk of kidney failure are considered for glucocorticoids, subsequent to consideration of clinical trial enrollment.^9^ Although systemic corticosteroids are used in clinical practice, they are not without side effects and the risks and benefits of immunosuppressive treatment should be considered carefully.^9^^,^^12^ Even with the option of immunosuppressive therapy, disease control remains difficult to achieve in a substantial proportion of patients. In the Intensive Supportive Care plus Immunosuppression in IgAN study, despite optimized supportive care with or without immunosuppression, approximately 50% of the 149-patient long-term follow-up cohort reached a composite end point of death, kidney failure, or a >40% decline in estimated glomerular filtration rate over a median follow-up period of 7.4 years.^13^ More recently, a targeted-release formulation of budesonide, administered in addition to optimized renin-angiotensin-aldosterone system inhibitors, has been approved as a novel gastrointestinal-targeted corticosteroid therapy for patients with primary IgAN at risk of kidney failure.^14^ Other therapies under clinical investigation include endothelin receptor antagonists; B-cell directed agents, such as those targeting B-cell activating factor and a proliferation-inducing ligand; and complement inhibitors.^15^

These novel therapies hold promise as corticosteroid-sparing treatments to reduce immune complex-driven kidney inflammation and damage, and a number of complement inhibitors have now entered late-stage clinical trials.^16^ Considering the evolving treatment landscape, this review examines current knowledge about the role of the complement system in IgAN and considers how targeted complement inhibitors have the potential to change clinical practice. The remaining knowledge gaps that should be addressed to guide clinical decision-making and optimize patient outcomes in this new treatment era will also be discussed.

### The Complement System and IgAN

#### The Complement System: A Brief Overview

The complement system plays a key role in innate immunity and acts as a modulator of the adaptive immune response, therefore being important for both immunosurveillance and maintaining tissue homeostasis.^17^^,^^18^ The system comprises an activation cascade involving approximately 50 soluble and cell-surface proteins, some activated by proteolytic cleavage.^18^ Complement system activation can be initiated via 3 pathways, namely the classical pathway, the lectin pathway (LP), and the alternative pathway (AP) (Figure 1^19^, ^20^, ^21^, ^22^, ^23^, ^24^, ^25^, ^26^).^27^^,^^28^ The classical pathway is activated by the interaction between C1q and immune complexes composed of immunoglobulin (Ig)M or IgG and antigen.^18^ The LP is activated when circulating mannan-binding lectin (MBL) binds to carbohydrate groups on the surface of pathogens. MBL-associated serine proteinase (MASP) 2 is essential for the activation of this pathway.^18^^,^^19^ The AP accounts for 80% of complement activity^17^ and, unlike the other pathways, it is constitutively active at low levels through spontaneous hydrolysis of C3, known as “tick-over.”^27^ Although each pathway has a different triggering mechanism, they all lead to the generation of C3 convertases, protein complexes that cleave C3 into C3a and C3b.^18^ The AP C3 convertase drives the AP amplification loop, which increases complement activity of all 3 pathways via the production of C3b.^28^^,^^29^ This process can rapidly amplify if not regulated, leading to activation of the terminal pathway and formation of the membrane attack complex (C5b-9). In addition, C3a and C5a anaphylatoxins are produced, which independently amplify inflammation and tissue damage.^18^

**Figure 1:**
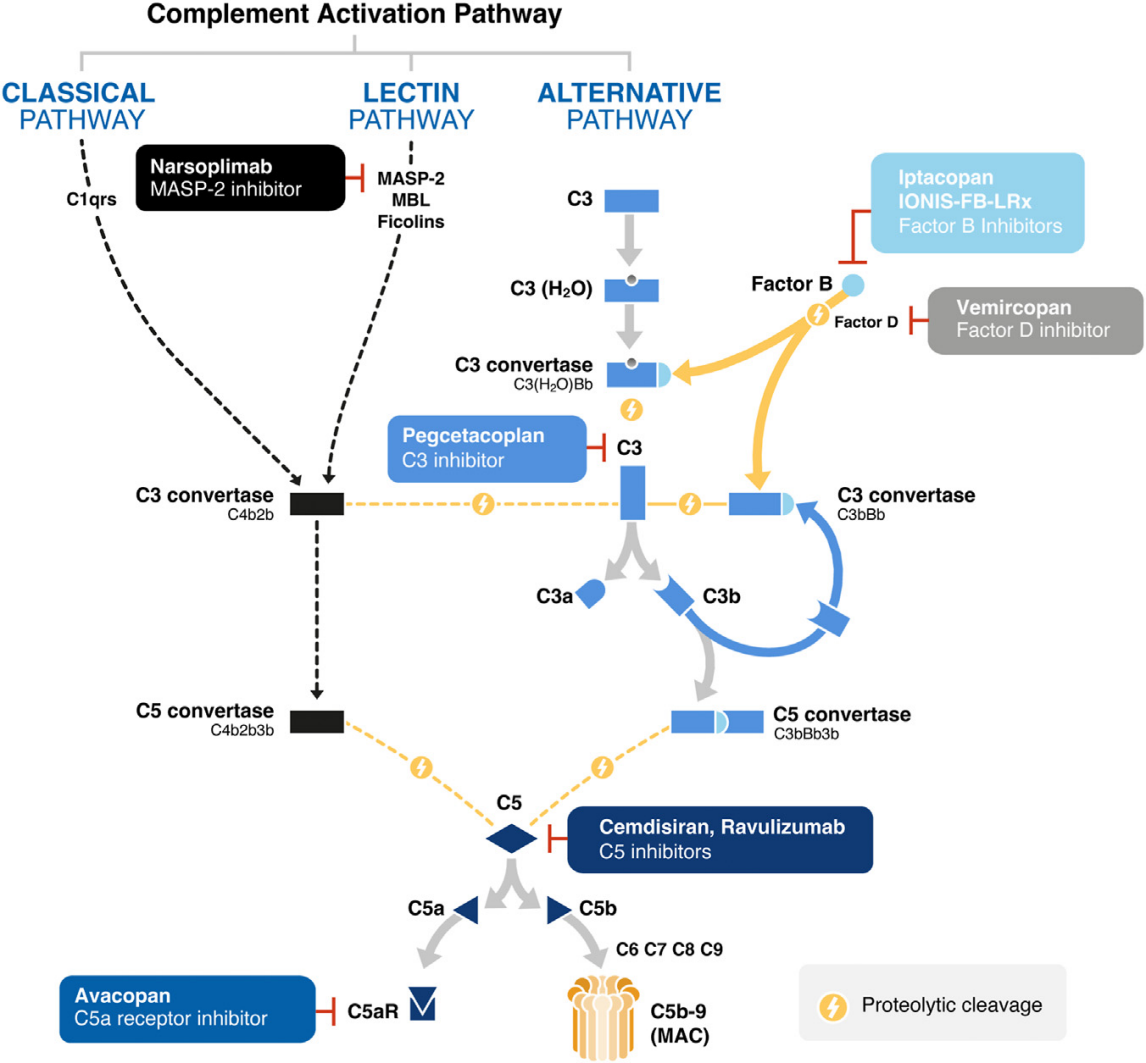
Complement system pathways and therapeutic targets for complement inhibitors in development for IgAN.^a^^19^, ^20^, ^21^, ^22^, ^23^, ^24^, ^25^, ^26^ IgAN, IgA nephropathy; MAC, membrane attack complex; MASP-2, mannan-associated lectin-binding serine protease-2; R, receptor; MBL, mannan-binding lectin. ^a^Investigational agents listed are in phase 2/3 development for the treatment of IgAN.

#### Role of the Complement System in IgAN Pathophysiology

Although understanding of the pathogenesis of IgAN continues to evolve, the autoimmune nature of the disease is considered to be driven by 4 “hits,” beginning with increased production of galactose-deficient IgA1 (Gd-IgA1, Hit 1) and the development of specific IgG or IgA1 autoantibodies targeting the hinge region of Gd-IgA1 (Hit 2; Figure 2).^18^^,^^30^ Activation of the complement system by immune complexes of Gd-IgA1 and anti-Gd-IgA1 autoantibodies (Hits 3 and 4), and subsequent triggering of glomerular inflammation and kidney injury, is recognized as an important contributing factor to the pathogenesis of IgAN.^18^^,^^30^ This is supported by a growing body of evidence suggesting a role for the AP and LP of complement in IgAN, as described in detail in several recent reviews.^6^^,^^18^^,^^27^^,^^31^

**Figure 2:**
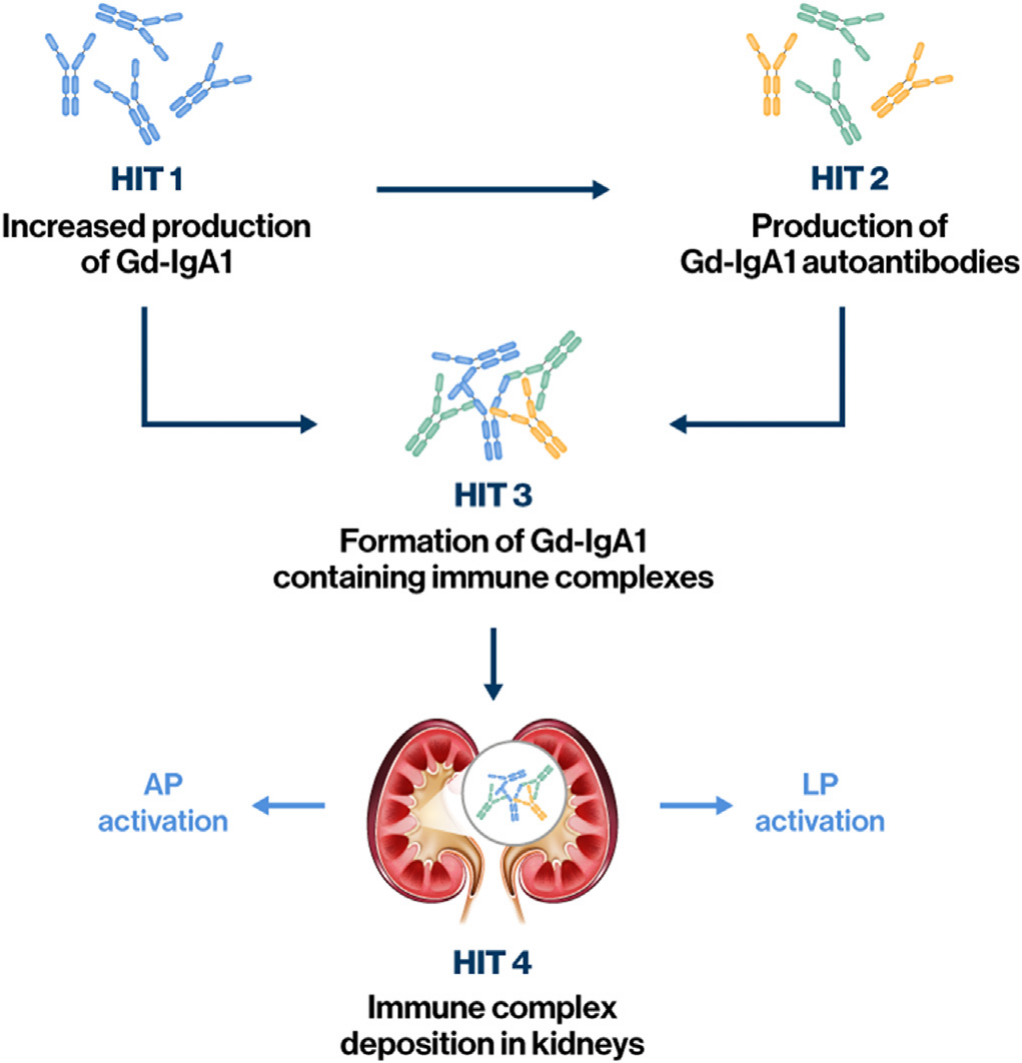
Summary of the 4-hit pathogenesis of IgAN and interaction with the complement system. AP, alternative pathway; Gd-IgA1, galactose-deficient immunoglobulin A1; IgAN, IgA nephropathy; LP, lectin pathway.

In kidney biopsy samples from patients with IgAN, evidence strongly indicates the involvement of the AP and LP as opposed to the classical pathway. Immunohistochemical findings report the mesangial deposition of C3 in up to 100% of patients, often accompanied by properdin, indicative of AP activation.^32^, ^33^, ^34^ Mesangial deposition of C4, particularly C4d, and MBL, is indicative of LP activity and has been reported in a proportion of patients with IgAN.^33^^,^^35^^,^^36^ Moreover, there is a link between glomerular AP and LP deposits and disease severity^36^, ^37^, ^38^; for example, C4d deposition has been linked to kidney failure.^39^, ^40^, ^41^

Proteins involved in regulating the AP, particularly factor H-related (FHR) proteins, have been implicated in IgAN pathogenesis, and genome-wide association studies point to a role of deletions in *CFHR1* and *CFHR3* in IgAN susceptibility.^42^ C3 and C3 breakdown products have been observed in circulating Gd-IgA1 immune complexes in patients with IgAN.^43^^,^^44^ In summary, overactivation of the AP and LP contribute to kidney inflammation and glomerular injury in IgAN, resulting in proteinuria, hematuria, and deterioration of kidney function.^6^

#### Complement Inhibitors: Clinical Evidence to Date

Several complement inhibitors are currently in development that target different parts of the complement system, including proteins in the LP (MASP 2 inhibitors), the AP (inhibitors of factor D and factor B, and factor B antisense oligonucleotide), and universal/common factors (inhibitors of C3 and C5, and C5 small interfering RNA).^15^^,^^20^^,^^21^ Therapeutic agents under evaluation in phase 2 or 3 studies in patients with IgAN include (Figure 1)^19^, ^20^, ^21^, ^22^, ^23^, ^24^, ^25^, ^26^: narsoplimab (a MASP 2 inhibitor),^19^ iptacopan (LNP023, a factor B inhibitor),^22^^,^^29^ vemircopan (a factor D inhibitor),^20^^,^^45^ pegcetacoplan (a C3 inhibitor),^23^ IONIS-FB-LRx (a factor B antisense oligonucleotide),^21^ cemdisiran (a C5 small interfering RNA),^15^^,^^24^ ravulizumab (an anti-C5 monoclonal antibody),^25^^,^^46^ and avacopan (a C5a receptor inhibitor).^26^^,^^45^

Complement inhibition in IgAN was first tested as salvage therapy, with the use of the anti-C5 monoclonal antibody eculizumab being reported in several case studies.^18^^,^^47^, ^48^, ^49^ Although initial improvements in kidney function were observed in some cases, treatment with eculizumab did not prevent eventual kidney failure. It has been acknowledged that the timing of treatment was sporadic and of short duration in these case studies.^6^ Terminal pathway inhibition with eculizumab is not included in the Kidney Disease: Improving Global Outcomes clinical practice guidelines for the treatment of IgAN,^9^ and it is considered unlikely that this treatment will enter clinical practice.^15^

Nonetheless, early data from novel agents indicate that targeting the complement system may be of clinical benefit in patients with IgAN. For example, inhibition of the LP via MASP 2 with narsoplimab reduced 24-hour urine protein excretion by 54% to 95% (*n* = 4) compared with baseline in a single-arm substudy of a phase 2 trial.^19^ Interim analysis of the subsequent randomized, vehicle-controlled substudy showed that narsoplimab treatment was well tolerated, with a median reduction in 24-hour urine protein excretion of 61.4% during a dose-extension phase (up to 54 weeks post-baseline; *n* = 8).^19^

The AP also holds promise as a therapeutic target in IgAN. Iptacopan, a selective inhibitor of factor B, was well tolerated in a randomized, double-blind, dose-ranging phase 2 study in patients with biopsy-confirmed IgAN.^50^ In patients treated with iptacopan 200 mg twice daily, up to a 40% reduction in urinary protein-to-creatinine ratio from baseline was observed, along with sustained inhibition of AP biomarkers. Both narsoplimab and iptacopan are being further evaluated in the ongoing phase 3 ARTEMIS-IgAN and APPLAUSE-IgAN studies, respectively.^22^^,^^51^

Among agents under investigation that target components of the terminal pathway, avacopan, a selective C5a receptor inhibitor, numerically improved urinary protein-to-creatinine ratio by up to approximately 50% in 3 of 7 patients in a phase 2, open-label pilot study including patients with IgAN on stable renin-angiotensin-aldosterone system inhibitors.^26^ Cemdisiran, a small interfering RNA that inhibits hepatic production of C5, has also been studied in a phase 2, randomized, double-blind, placebo-controlled trial. Overall, 31.8% of patients treated with cemdisiran (*n* = 22) achieved ≥50% reduction in 24-hour urinary protein-to-creatinine ratio at week 32, compared with 12.5% of placebo-treated patients (*n* = 9).^52^

There is a strong potential for complement inhibitors to improve kidney outcomes in patients with IgAN; however, the completion of large randomized controlled trials is needed to confirm efficacy and potentially identify optimal patient populations for each targeted treatment.

### Biomarkers in the Era of Complement Inhibitors

Given the number of potential candidates under investigation for complement pathway inhibition in patients with IgAN, it is likely that complement inhibitor therapeutics will be introduced into the IgAN treatment paradigm in the near future. In conjunction with this, there will be a need for biomarkers that provide a strategy to guide personalized patient management by offering the ability to identify which complement pathways are active as well as the level of activity.

#### Histopathologic Markers of Complement Activation

Diagnosis of IgAN relies on a kidney biopsy to identify predominant or codominant IgA deposits within the glomerular mesangium as the defining feature.^9^ The Oxford Classification MEST-C (mesangial [M] and endocapillary [E] hypercellularity, segmental glomerulosclerosis [S], tubular atrophy/interstitial fibrosis [T], cellular/fibrocellular crescents [C]) histopathologic scoring system is routinely used as part of diagnosis and for predicting risk of disease progression.^9^ It is hoped that the type and amount of complement deposition could determine if a patient with IgAN would benefit from complement inhibition and, if so, which complement pathway.^27^ To date, multiple immunofluorescence and immunohistochemical stains for the detection of complement proteins in kidney biopsies are available, as summarized in Table 1.^18^^,^^27^^,^^53^, ^54^, ^55^, ^56^, ^57^ Currently, standard practice for native kidney biopsies typically includes staining for C1q and C3c by immunofluorescence microscopy, with optional or ancillary testing for C3d and C4d by immunofluorescence or immunohistochemistry.^58^

**Table 1:** Glomerular markers of complement activity in native kidney biopsies in patients with IgAN^18^^,^^27^^,^^53^^,^^54^

Complement Protein(s)	Pathway			Applicability in Clinical Practice for the Evaluation of a Kidney Biopsy in IgAN^a^
	Classic	Lectin	Alternative	
C1q^b^	+	−	−	+++
	Pathway(s) if C1q staining is negative			
C2	−	+	−	++
C3a	−	+	+	+
C3aR	−	+	+	+
C3b, iC3b, C3c	−	+	+	+++
C3d	−	+	+	+
C3dg	−	+	+	+
C4d	−	+	–	+++
C5b-9	−	+	+	++
FH	−	−	+	++
FHR1	−	−	+	++
FHR5	−	−	+	++
Properdin	−	−	+	+
MBL	−	+	−	+
MASP-2, MASP-1/-3	−	+	−	+
L-ficolin	−	+	−	+

AP, alternative pathway; C3aR, complement component 3a receptor; CP, classical pathway; FHR5, factor H-related protein 5; IgAN, IgA nephropathy; LP, lectin pathway; MBL, mannan-binding lectin; MASP, MBL-associated serine proteinase.

a Applicability of immunohistochemical and immunofluorescent stains in clinical practice is scored using + (applicable in some circumstances), ++ (applicable), and +++ (highly applicable) (based on authors’ expert opinion).

b C1q staining is generally absent in kidney biopsy specimens of patients with IgAN, though C1q deposition has been reported in some studies.^55^, ^56^, ^57^

Recent studies have investigated the prognostic value of C3 deposition in IgAN and examined the relationship with the Oxford MEST-C score.^59^, ^60^, ^61^, ^62^ Findings suggest that high intensity of glomerular C3 staining, in addition to the MEST-C score, may predict a high risk of IgAN progression.^59^, ^60^, ^61^, ^62^ However, there remain technical challenges associated with quantification of immunofluorescence, including background staining levels and subjective interpretation of results. The clinicopathological significance of glomerular deposition of varying C3 breakdown products (C3b, C3c, iC3b, and C3d) is an area of investigation^18^ and has implications regarding interpretation of C3 staining intensity. For instance, C3c is a biologically inactive peptide that dissociates rapidly after proteolytic cleavage, whereas C3d remains covalently bound. Therefore, glomerular C3c deposition may indicate active, inflammatory activity in IgAN and may be decreased during the inactive, chronic phase of disease, during which C3d deposition remains present.^63^ Glomerular staining for FHR5 may also correlate with disease progression in IgAN, in addition to serving as a marker of AP activation.^53^

In terms of LP proteins, mesangial MBL and C4d deposition (which indicates LP activity in the absence of positive C1q staining) have been reported to correlate with worse outcomes in IgAN.^18^^,^^40^^,^^41^^,^^64^ In a pooled multivariate analysis, glomerular C4d staining was a strong independent risk factor for kidney failure in patients with IgAN, even in those at early stages of disease. Of note, the same analysis reported a higher proportion of glomerular C4d staining in studies conducted in Asian populations; however, interstudy variation in definitions of C4d positivity and disease severity among the included populations warrants consideration.^41^ Regarding the terminal pathway, few studies have explored C5b-9 deposition in IgAN, but a retrospective analysis of kidney biopsies suggests a role for C5b-9 deposition in the development of tubulointerstitial lesions.^65^ Finally, inflammatory cell infiltration in the kidney is a marker of ‘active’ IgAN, with higher cell counts of glomerular CD68+ macrophages associated with endocapillary hypercellularity.^66^^,^^67^ In a study of Chinese patients with IgAN who were at high risk of progression, the intensity of glomerular CD68+ macrophage staining increased the probability of response to immunosuppressive therapy.^67^ Because complement activation contributes to glomerular inflammation, such an approach may merit investigation to identify patients who might benefit from complement inhibition and as a tool to monitor treatment responses.

Further research into glomerular staining for complement proteins is warranted, especially to ascertain the predictive value of these biomarkers alongside novel therapies currently in development. A biomarker that can reliably predict or assess treatment response has considerable potential to change the future of care. Nonetheless, though information from diagnostic kidney biopsies is of clinical value, repeat biopsies to assess treatment efficacy over time are not always practical or ethically justified; therefore, noninvasive methods are also needed.

#### Noninvasive Biomarkers of Complement Activity in IgAN

Although serological and urinary markers of complement activity have been investigated, none are validated for clinical use or commercially available for IgAN*.* The plasma Gd-IgA1:C3 ratio is one of the most widely studied markers and has been independently associated with progression to kidney failure in cohort studies of patients with IgAN.^68^^,^^69^ Although this ratio reflects both elevated circulating galactose-deficient IgA1 (Gd-A1) and decreased serum C3 levels in an individual, serum Gd-A1 may be a key driver because serum C3 levels are often within the normal range in patients with IgAN.^6^^,^^27^ Serum IgA:C3 ratio has also been investigated as a diagnostic tool,^70^^,^^71^ with a high ratio reportedly an effective predictor of an IgAN diagnosis, in particular in patients with proteinuria ≤1 g/d.^70^

Serological biomarkers of AP activation associated with IgAN severity include elevated levels of FHR1, FHR5, and factor Ba.^42^^,^^72^^,^^73^ Patients with IgAN were shown to have higher levels of plasma factor Ba than healthy controls in 1 study; factor Ba levels were also shown to correlate with Gd-IgA1 concentration and disease activity.^73^ In a subsequent study, plasma levels of Gd-IgA1 and factor Ba were shown to independently predict higher T scores of the Oxford MEST-C classification.^74^ FHR1 and FHR5 antagonize the inhibitory role of factor H, a negative regulator of the AP.^72^ The role of factor H deregulation in IgAN progression has been reported by independent studies associating high serum FHR1:factor H ratio with a progressive decline in kidney function.^42^^,^^72^

Regarding urinary biomarkers, increased urinary excretion of the AP proteins factor H and properdin has been reported in patients with IgAN compared with healthy controls. In contrast, levels of complement receptor 1, a membrane-bound regulator that acts on C3b, have been reported to be significantly lower.^75^ Interestingly, elevated levels of urinary factor H have been associated with a worse prognosis and kidney injury in IgAN.^75^, ^76^, ^77^ Factor H functions as a key inhibitor of the AP, and it has been suggested that increased levels in the urine may reflect intrakidney complement synthesis, potentially as a compensatory mechanism to prevent overactivation of the local complement system^62^^,^^75^^,^^78^; although the source of urinary factor H in patients with IgAN remains to be elucidated.^76^^,^^78^

Noninvasive markers of LP activation have also been investigated; however, both low and high MBL levels have been associated with disease severity, highlighting the complexity of IgAN pathophysiology.^79^ Other LP abnormalities include increased plasma M-ficolin, L-ficolin, MASP-1, and MBL-associated protein 19 levels and lower MASP-3 levels in patients with IgAN compared with healthy controls.^53^

In terms of the terminal pathway markers, urinary C5b-9 levels have been explored as a prognostic biomarker in IgAN and have been shown to correlate positively with proteinuria.^75^^,^^80^ As a note of caution, increased urinary excretion of complement proteins has been shown to correlate with levels of proteinuria in several kidney disorders and it has been suggested that this might be related to chronic injury and proximal tubule dysfunction as opposed to active glomerular disease^78^^,^^81^; therefore, studies to clarify the pathological mechanisms underlying urinary C5b-9 and factor H excretion in IgAN are warranted.

### The Complement Inhibitor Era in IgAN: Remaining Challenges

Although the future availability of complement inhibitors in clinical practice is likely to make an important contribution to the personalized management of patients with IgAN, several key questions and challenges remain, as summarized in Figure 3.

**Figure 3:**
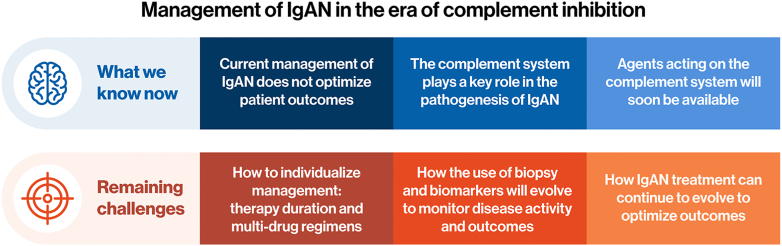
Remaining challenges in the management of IgAN. IgAN, IgA nephropathy.

#### Further Unraveling of the Role of Complement in IgAN Progression

Though histological evidence of complement activation in IgAN at the time of the diagnostic biopsy has been reported,^18^^,^^27^ it is unknown if the level of activation changes during the course of the disease. A single pathogenic mechanism for kidney inflammation and injury in IgAN is unlikely, and the variation in complement deposition reported in kidney biopsies indicates heterogeneity in the contribution of the AP and LP.^27^ Furthermore, notable ethnic variation in histopathological lesions and genetic associations in IgAN exist.^82^ While the activators for the AP and LP have been previously documented,^18^ further research is required to clarify what determines either AP or LP activation and to elucidate whether both pathways may be activated in the same patient at different times. Studies of complement activation in the kidney have focused on the pathways in isolation^83^; understanding whether there is a dominant pathway driving glomerular inflammation, as well as gaining insights into the potential impact of complement inhibition on glomerular versus tubulointerstitial injury, is therefore crucial to guiding personalized treatment approaches.

#### Identifying Targeted Candidates for Complement Inhibition

As discussed earlier, several inhibitors are in clinical development that target different components of the complement system. Their successful implementation in clinical practice will require the use of biomarkers to inform patient selection and monitoring of response.^83^ Continued research into glomerular staining of complement proteins therefore remains an important area of focus; however, high levels of background staining present a challenge, which may limit utility and reproducibility. Immunofluorescence and immunohistochemical stains to assess the presence and contribution of complement protein deposits will require optimization, standardization, and widespread implementation as part of the evaluation of diagnostic kidney biopsies in patients with IgAN. Whether current stains are sufficient to guide clinical decision-making warrants further investigation and, ideally, validation of a “complement panel”. Such a panel could help confirm complement activation, determine the extent of activation, and provide information on the respective contributions of the AP and LP in each patient.^83^ Nonetheless, complement activation in IgAN is not a static process; therefore, though histopathological analyses of a kidney biopsy provide valuable insights, they inform only on the contribution of complement during a “snapshot” of disease.

Overactivation of the complement system causes kidney inflammation and glomerular injury^84^; serological and urinary biomarkers of complement activity therefore hold promise as noninvasive methods for identifying patients with IgAN who might benefit from targeted complement inhibitors. Despite recent advances, a need remains to develop and commercialize sensitive and specific noninvasive biomarkers of AP, LP, and terminal pathway activity. Practical considerations also exist when assessing serological biomarkers; to maintain function of complement components and avoid *in vitro* complement activation, serum samples should be kept cold and stored frozen at −80 °C, preferably within 4 hours of sampling. In addition, for analyses of individual complement components and activation products, ethylenediaminetetraacetic acid–plasma should be prepared because the use of ethylenediaminetetraacetic acid as a chelating agent prevents further activation of complement by the C1 complex and C3 convertases.^85^

#### Translating Current Understanding of the Complement System to Inform Clinical Practice

Current guidelines recommend the use of the Oxford Classification MEST-C score and the International IgAN Prediction Tool for prognostication in IgAN.^9^ At present, the relevance of these tools in guiding treatment decisions is unclear and markers of complement activity are not incorporated.^9^ In light of the therapeutic targeting of complement as a future treatment approach in IgAN, further efforts to validate markers of complement activity are needed, with their possible incorporation into available prognostic tools. Monitoring of treatment responses in IgAN is currently limited to nonspecific measures, including proteinuria, estimated glomerular filtration rate, and hypertension. In this chronic disease setting, biomarkers to assess response to complement inhibition, including potential achievement of remission, will be necessary to guide treatment duration and monitor response to therapy.^83^

Of note, safety data on complement inhibitors are needed before their adoption in clinical practice. The risk of infection from encapsulated bacteria is the principal safety concern.^18^ Although complement-mediated immunity is unlikely to be fully inhibited, vaccinations for common encapsulated bacteria will likely be required.

#### The Way Forward

Data from large-scale clinical trials and patient registries will be crucial to unravel further the role of the complement system in IgAN pathophysiology and to address the remaining challenges. Until such data are available, there is insufficient evidence to predict which patients will respond to complement inhibitor therapies targeting different pathways. In addition to evidence from clinical trials, experience from other diseases where complement inhibitors are either approved or being studied, for example, C3 glomerulopathy and atypical hemolytic uremic syndrome,^16^^,^^86^ may provide valuable insights. Taking another approach, multiomics-based strategies also hold promise^87^^,^^88^; transcriptomic and miRNomic analyses of tissue from kidney biopsies may do much to guide biomarker identification over the next decade. Although studies involving these techniques are still in the early stages, it is hoped that the results will offer valuable information about disease prognosis in IgAN, supporting patient selection and treatment decisions.

### What Does the Future Look Like in IgAN?

Treatment options and management pathways for patients with IgAN are evolving; however, there remain areas of uncertainty, including an incomplete understanding of disease pathophysiology.^15^ Several complement inhibitors have entered late-stage clinical trials in IgAN.^16^ Complement inhibitors may provide steroid-sparing control of kidney inflammation and damage while waiting for the development of future and complementary therapies that target upstream production of the pathogenic Gd-IgA, Gd-IgA-specific immunoglobulins, and the immune complexes they both form. It will be crucial to gain an understanding of the efficacy and safety of these treatments, particularly when under consideration for use as part of long-term management. Determining the relationship between the degree of inhibition of the targeted complement pathway in question and response to treatment will also be important. Because of the complex underlying mechanisms of disease, it is unlikely that a single therapy will be effective in all patients with IgAN. Future management should reflect individualized treatment approaches implemented in other specialties, such as oncology and cardiology, a key part of which will be to match treatment with the individual patient, in addition to the established standard of care. Here, advances in reliable, noninvasive biomarkers of disease activity and remission will be critical to shaping clinical practice. Use of biomarkers to facilitate personalized therapy will likely help address the issue of cost as the field incorporates more targeted therapies into treatment paradigms. Finally, in the long-term, multidrug approaches may need to be evaluated as multiple novel agents become pillars in the treatment of patients with IgAN.

In conclusion, the long wait for a new era of IgAN management may soon be over, with novel treatments and biomarkers soon to be at our disposal to prevent progression, reduce the burden of disease, and increase life expectancy in patients with IgAN.

## Disclosure

VT reports consulting fees from AstraZeneca, Boehringer-Ingelheim, Calliditas Therapeutics, Novartis, Omeros and Otsuka and is a member of the executive committee of the International Society of Nephrology. JR has received consulting fees from Travere Therapeutics and Calliditas Therapeutics. VC reports research grants from NIH. JB reports research grants from Argenx, Calliditas Therapeutics, Chinook Therapeutics, Galapagos, GlaxoSmithKline, Novartis, and Travere Therapeutics and is a medical/scientific advisor for Alnylam Pharmaceuticals, Argenx, Astellas Pharma, Biocryst Pharmaceuticals, Calliditas Therapeutics, Chinook Therapeutics, Dimerix, Galapagos, Novartis, Omeros, Travere Therapeutics, UCB, Vera Therapeutics, and Visterra.
